# Prevalence of Intermediate Syndrome among Admitted Patients with Organophosphorous Poisoning in a Tertiary Care Hospital

**DOI:** 10.31729/jnma.4569

**Published:** 2019-10-31

**Authors:** Ananta Bhakta Uprety, Binod Pantha, Lochan Karki, Suresh Prasad Nepal, Milan Khadka

**Affiliations:** 1 Department of Medicine, Bir Hospital, Kathmandu, Nepal; 2 Department of General Practice, Bir Hospital, Kathmandu, Nepal

**Keywords:** *gastric lavage*, *intermediate syndrome*, *organophophorus poisoning*

## Abstract

**Introduction:**

Organophosphorous poisoning is a common problem prevalent in Nepal. Intermediate syndrome is a common clinical feature seen among the patients those have ingested poison. There is a scarcity of data related to intermediate syndrome and other general complications in patients with organophosphorous poisoning in context of Nepal. This study was carried out to observe the prevalence of intermediate syndrome and the general complications of oraganophosphorus poisoning among admitted patients in a tertiary care hospital.

**Methods:**

This was a descriptive cross-sectional study conducted at a tertiary care hospital from April 2008 to June 2009 after ethical approval was from Institiutional Review Board of tertiary care hospital. Forty four patients with history of ingestion of organophosphorus poisoning within 24 hours were included in our study through convenience sampling. Clinical examinations were done to look for Intermediate syndrome. Data was entered in Statistical Package for Social Sciences and point estimate at 95% of CI was calculated along with frequency and proportion for binary data.

**Results:**

Out of 44 patients, features of intermediate syndrome were seen in 40 (90.9%) at 95% of CI (84.2-97.6) patients in the study. The frequency of intermediate syndrome signs like weakness of neck flexion, inability to sit up and swallowing difficulty were seen among the patients. Complications like pneumonia 4 (9.09%), hyponatremia 3 (6.8%), hypokalemia 1 (2.27%) and bradycardia 1 (2.27%) were seen in the study. Mortality seen in the study was 2 (4.5%) among the admitted patients.

**Conclusions:**

Prevalence of intermediate syndrome was higher compared to other studies done in similar settings. Complications like pneumonia, hyponatremia, hypokalemia and bradycardia were seen among the patients.

## INTRODUCTION

Organophosphorus compounds are used as pesticides, herbicides and chemical warfare agents in the form of nerve gases. Intermediate Syndrome (IMS) develops in 24-96 hours after the ingestion of OP poison, involves the symptoms of weakness of proximal muscle groups, neck, trunk, cranial nerve palsies and decreased tendon reflexes and may require mechanical ventilation.^1^ In our community, poisons consumed are commonly organophosphorous compounds; aluminium phosphide. Gastric lavage is useful within the first four hours of poison ingestion except in tricyclic antidepressants, salicylates and morphine poisoning and in an unconscious patients where lavage can be done several hours later.

In Nepal OP poisoning is very common frequently in the young female population mostly due to domestic violence. No adequate studies are there regarding the complications in the Nepalese context.

The aim of this study is to see the prevalence of intermediate syndrome and other complications associated with Organophosphorus poisoning.

## METHODS

This was a descriptive cross-sectional study conducted at Bir Hospital from April 2008 to June 2009. Ethical approval was taken from Institutional Review committee of Bir hospital. Convenience sampling was done and sample size calculation was calculated using the following formula,

n = Z^2^ × (p × q)/e^2^

where,

n = sample size
Z =1.65 for Confidence Interval of 90%
p = prevalence, 80% (educated guess)
q = 1-p
e = margin of error = 10%

Total sample required for the study was taken to be 44.

Patients who came to the emergency department with a history of ingestion of OP poison within 24 hours and admitted in the inpatient department were included in the study. Patients with other concomitant poisoning and pregnancy were excluded.

The required information was gathered as per written in the Performa. Informed consent was taken from the patient or from the patient’s party. Ingestion of poison was identified by clinical history, tracing the content of the poison (bottle), smell, signs and symptoms. Gastric decontamination by lavage was done until 6 hours from the time of ingestion of poison. The gastric lavage sample was sent to National Forensic Science Laboratory for the determination of the type of poison. Signs and symptoms of OP poisoning were recorded and treatment with atropine and PAM was started to all patients. Clinical examinations were done to look for IMS-like weakness of neck flexion, inability to sit up, ophthalmoparesis, slow eye response, facial weakness, swallowing difficulty, limb weakness (proximal more than distal), areflexia, respiratory insufficiency and each of these symptoms and signs were recorded of patients along with any complications present.

Data was entered in SPSS and point estimate at 95% of CI was calculated along with frequency and proportion for binary data.

## RESULTS

**Figure 1:**
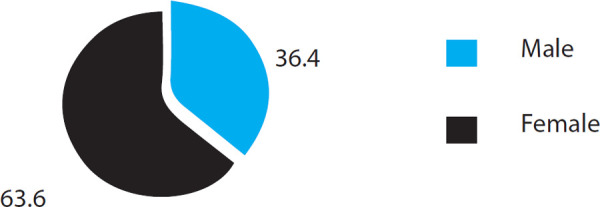
Sex distribution among OP poison patients.

Out of 44 patients, 28 (63.6%) were females and 16 (36.4%) were males (Figure 1).

**Table 1:** Age distribution in OP poisoning cases.

	Age (in years)	20-30	31-40	41-50
	Male	9	3	4
Gender	Female	18	9	1

The most frequently observed clinical feature of IMS was weakness of neck flexion among 40 (90%) followed by inability to sit up in 39 (88%), swallowing difficulty in 34 (77%), facial weakness in 27 (61%), limb weakness in 16 (36%) and opththalmoparesis in 15 (34%). Slow eye response and areflexia are less observed, however respiratory insufficiency was observed only in 2 patients with IMS (Table 2).

**Table 2:** Intermediate syndrome in OP poisoning patients.

Parameters	Patients with OP poisoning n (%)
Weakness of neck flexion	40 (90.9%)
Inability to sit up	39 (88.6%)
Swallowing difficulty	34 (77.2%)
Facial weakness	27 (61.3%)
Limb weakness (proximal >distal)	16 (36.3%)
Ophthalmoparesis	15 (34.09%)
Slow eye response	12 (27.2%)
Areflexia	10 (22.7%)
Respiratory insufficiency	2 (4.54%)

The most frequent complication was pneumonia accounting for 4 (9.09%) followed by hyponatremia 3 (6.81%), hypokalemia 1 (2.27%) and bradycardia 1 (2.27%) seen among patients (Table 3).

**Table 3:** Morbidity (complications and hospital stay) in OP poisoning.

Complication	Patients with OP poisoning n (%)
Pneumonia	4 (9.09%)
Hyponatremia	3(6.81%)
Hypokalemia	1 (2.27%)
Bradycardia	1 (2.27%)

In total, mortality was found among 2 (4.5%) patients with organophosphorus poisoning.

## DISCUSSION

Organophosphates are widely used in agriculture worldwide and are a common cause of poisoning that continues to result in significant fatalities. This is because of free availability of pesticides and its over-the-counter sale. Certain factors, e.g. severely intoxicated patients, involvement of drugs that delay absorption and gastric emptying (e.g. tricyclic antidepressant), ingestion of substances which require metabolic activation before becoming toxic (e.g. paracetamol, methanol, ethylene glycol, and some organophosphate insecticides), ingestion of large quantities of toxic drugs, absence of bowel sounds on physical examination indicate the possible usefulness of gastric lavage even as long as 4 to 6 hours after ingestion. For the study, as OP poisoning is a potentially lethal situation with relatively high mortality, gastric lavage has been considered up to 6 hours after ingestion.

In our study mean age among both groups of patients with or without gastric lavage was found to be 31 years. Similarly, in a study by Agrawal SB, the maximum number of cases was in the age group of 21 to 30 years.^3^ Another study done by Poojara L, et al. also showed mean age of 30 years among the OP poisoning cases. The prevalence of OP poisoning in the young age group may be due to the increase in stress because of unemployment, poverty and conflicting relationships in young couples.^4^

Females were found to have higher proportion of poison consumption rate accounting for 63.6% vs. 36.4% in males. Male:Female ratio was 1:1.75 in present study. Similar results were found in some other studies, where females were found to have higher of poison consumption rate.^2^ The features of IMS were seen in 40 cases out of 44 (90.9%) in the present study. The incidence of IMS in different studies has been reported to be between 20-68%. The reported incidence of IMS ranges from 7.7% to as high as 84%.^5-11^ In our study, the incidence of IMS was found to be higher compared to other studies. The higher incidence of IMS in our study could be due to the inclusion of patients who had even a single symptom of intermediate IMS e.g. weakness of neck flexion. The late presentation of patients of OP poisoning to the hospital could be the reason for high incidence of IMS in our study.

The most frequent observed clinical features of IMS were weakness of neck flexion, 90.9% followed by inability to sit up 88.6%, swallowing difficulty 77.2%, facial weakness 61.3%, limb weakness 36% and opththalmoparesis 34%. Slow eye response and areflexia were less observed, and respiratory insufficiency was observed only in 2 patients with IMS. Clinical features of IMS were observed more among patients who underwent gastric lavage than in patients who didn’t. The respiratory insufficiency was observed only in patients with gastric lavage.

In 1987, workers from Sri Lanka described IMS, which included signs of paralysis appearing in 10% to 40% of patients that occurred 24 to 96 hours after exposure, i.e. after admission and before the delayed neurotoxicity.^12^

IMS develops 12-96 hrs after exposure and reflects the prolonged action of acetylcholine on the nicotinic receptors. The clinical features are muscular weakness in ocular, neck, bulbar, proximal limb and respiratory muscle with occasional dystonic posturing requiring mechanical ventilation in an ICU for several days. Cranial nerve palsies are common. The risk of mortality is due to the associated respiratory depression. The sensory functions characteristically remain normal and full recovery is evident in 4 to 18 days. It has been commonly associated with organophosphorous compounds like diazinon, dimethoate, methyl parathion, methamidaphe, monocrotophos, fenthion and ethyl parathion.^13^ Despite its common occurrence, data on the risk factors of IMS, early diagnosis and prediction have remained elusive. Commonly used tests such as levels of plasma cholinesterase correlate poorly with the onset of IMS.^14^

Mortality due to IMS was found in 4.5%. In Sri Lanka, about 10,000 to 20,000 are admitted to hospital for organophosphorus poisoning each year. Among cases, at least 10% died.^15^ Case mortality across the developing world is commonly greater than 20%.^16^ The study conducted in Multan from 1996 to 2000 showed 370 patients of OP poisoning with a mortality rate of 15%. Although, complications were seen more frequently in the gastric lavage group, early lavage was associated with lower mortality rate. In present study, complications occurred more common among patients with gastric lavage, and the most frequent complication was pneumonia accounting 11.5% followed by hyponatremia, hypokalemia, bradycardia at the time of discharge, 3.8% in each among patients with gastric lavage. However, hyponatremia was most frequent complication occurred among patients without gastric lavage accounting 11.1% followed by pneumonia i.e. 5.5% and no cases of hypokalemia and bradycardia observed in the said group.

Pneumonia was observed among the patients with little higher level of serum cholinesterase level (1000 U/L to 1700 U/L). Hyponatremia, hypokalemia and bradycardia at the time of discharge were found among the patients with low level of serum cholinesterase level (200 U/L to 1000 U/L). Similar types of complications were found in many studies. A study from Pakistan by Saleem Faiz, et al. mentioned about the acute complications like fits in 16.66%, bradycardia in 10% and hyperglycemia in 5% patients.^17^ Acute complications similar to this study were episodic convulsions developed in 16.66% patients, while 6.66% patients developed profuse diarrhea, and severe bradycardia was seen in 10% patients, hypotension in 10% patients, whereas 5% patients developed hyperglycemia and 1.66% patients developed acute renal failure in a study from Kashmir, India.^18^ Acute complications included hypokalemia in 40.9%, respiratory failure in 23.5%, hyponatremia in 23.5%, non-cardiogenic pulmonary edema in 18.26%, acute kidney injury in 11.3%, hypernatremia in 9.6%, mixed acidosis in 6.1%, metabolic acidosis in 5.2% and respiratory acidosis in 2.6%.

These were found in study done in Nepal which support our results regarding the complications of OP poisoning.^19^ Limitation of this study is small sample size due to which result could have been exaggerated or less accurate. Also, long term follow-up of the patients were not there so that remote complications were not observed.

## CONCLUSIONS

Prevalence of Intermediate Syndrome was higher compared to other studies. Clinical signs of IMS were seen in most of the cases where it occurred from day 2 to day 5. Complications like pneumonia, hyponatremia, hypokalemia, and bradycardia were more prevalent among the group of patients who underwent gastric lavage.

## Conflict of Interest:

None.
